# Large-Scale Analysis of Gene Expression Data Reveals a Novel Gene Expression Signature Associated with Colorectal Cancer Distant Recurrence

**DOI:** 10.1371/journal.pone.0167455

**Published:** 2016-12-09

**Authors:** Nehad M. Alajez

**Affiliations:** Stem Cell Unit, Department of Anatomy, College of Medicine, King Saud University, Riyadh, Kingdom of Saudi Arabia; Second University of Naples, ITALY

## Abstract

Colorectal cancer (CRC) is the fourth-ranked cause of cancer-related deaths worldwide. Despite recent advances in CRC management, distant recurrence (DR) remains the major cause of mortality in patients with preoperative chemotherapy and radiotherapy, underscoring a need to precisely identify novel gene signatures for predicting the risk of systemic relapse. Herein, we integrated two independent CRC gene expression datasets: the GSE71222 dataset, including 26 patients who developed DR and 126 patients who did not develop DR, and the GSE21510 dataset, including 23 patients who developed DR and 76 patients who did not develop DR. Our data revealed 37 common upregulated genes (fold change (FC) ≥ 1.5, *P* < 0.05) and three common downregulated genes (FC ≤ 1.5, *P* < 0.05) between DR and non-recurrent patients from the two datasets. We subsequently validated the upregulated gene panel in the Cancer Genome Atlas CRC datasets (379 patients), which identified a five-gene signature (*S100A2*, *VIP*, *HOXC6*, *DACT1*, *KIF26B*) associated with poor overall survival (OS, log-rank test *P*-value: 1.19 × 10^−4^) and poor disease-free survival (DFS, log-rank test *P*-value: 0.002). In a Cox proportional hazards multiple regression model, the five-gene signature and tumor stage retained their significance as independent prognostic factors for CRC DFS and OS. Therefore, our data identified a novel DR gene expression signature associated with worse prognosis in CRC.

## Introduction

Colorectal cancer (CRC) is one of the most prevalent types of cancers and is currently ranked as the fourth leading cause of cancer-related deaths globally, and the third leading cause of death in the United States in both men and women [1, 2]. The 5-year survival rate for CRC patients with a localized tumor is approximately 90%, which declines to 70% for patients with regional disease, and to 12% for patients with metastatic disease [2]. Multiple molecular alterations occur during CRC development and progression. Therefore, the identification of clinical and pathological parameters that can accurately predict the prognosis of patients with CRC has been a daunting task. Some of the factors to consider for predicting the risk of systemic relapse include the differentiation status of the tumor, depth of tumor invasion, and vascular and perineural invasion [3, 4]. Over the past several years, numerous molecular signatures have been identified for CRC prognosis [5–7]. However, one major problem with many of the established molecular signatures for CRC relapse is the lack of validation across different groups and platforms. Therefore, large-scale analysis of multiple gene expression datasets might lead to the identification of more representative gene expression signatures associated with CRC relapse. Herein, we integrated three independent CRC gene expression datasets retrospectively, which led to the identification of a novel five-gene signature associated with CRC systemic relapse.

## Materials and Methods

### Patient information and data analysis

The current study was conducted on three different CRC cohorts: (1) the National Center for Biotechnology Information Gene Expression Omnibus (GEO) GSE71222 dataset, which included 26 patients who developed distant recurrence (DR) and 126 patients who did not develop DR; (2) the GSE21510 dataset, which included 23 patients who developed DR and 76 patients who did not develop DR; and (3) The Cancer Genome Atlas (TCGA) CRC dataset, which included a total of 379 CRC patients. Interrogation of the TCGA dataset was conducted as previously described [8–10]. The relationship of gene expression patterns with patient survival in the TCGA database was queried using the cBioportal database with the formula GENE: EXP > 0, where GENE represents a query gene. The clinical characteristics for the TCGA dataset are shown in Table 1. The clinical characteristics for the GSE71222 and GSE21510 datasets have been described previously [11, 12].

**Table 1:** The Cancer Genome Atlas CRC dataset patient and tumor characteristics.

	N = 379	%
**Age, years**		
Median age	66	
Range	31–90	
**Gender**		
Male	206	54.4
Female	168	44.3
Unknown	5	1.3
**Overall survival, months**		
Median	22.04	
Range	0–147.9	
**Disease-free survival, months**		
Median	20.27	
Range	0–147.9	
**Stage**		
I	56	14.8
II	135	35.6
III	112	29.6
IV	52	13.7
NA	24	6.3

### Microarray data analysis

The GSE71222 and GSE21510 raw gene expression datasets were retrieved from the GEO and were imported into GeneSpring 13.0 software (Agilent Technologies, Palo Alto, CA, USA). Raw data were subsequently normalized using the percentile shift, and a 1.5 fold-change (FC) cutoff and *P* < 0.05 were used to determine significantly changed transcripts between groups [13].

### Statistical analysis

Kaplan-Meier survival curve comparison was conducted using the log-rank test, and a *P*-value of ≤0.05 was considered statistically significant. The Cox proportional hazards multiple regression model was used to identify the independent prognostic factors and to correct the effect of potential confounding variables, such as gender (male vs female), age (> 65y vs < 65y), tumor stage (stage 3/4 vs stage 1/2), and of cancer type (colon adenocarcinoma vs rectal adenocarcinoma vs mucinous adenocarcinoma of the colon and rectum) on OS and DFS using MedCalc 16.8.4 (MedCalc, Mariakerke, Belgium). Pathway analyses were conducted using DAVID functional annotation and clustering bioinformatics tool, as described in our previous reports [14, 15]. Statistical analyses and graphing were performed using Graphpad Prism 6.0 software (Graphpad Software, San Diego, CA, USA).

## Results

### Generation of a gene expression panel associated with risk of DR

**Fig 1:**
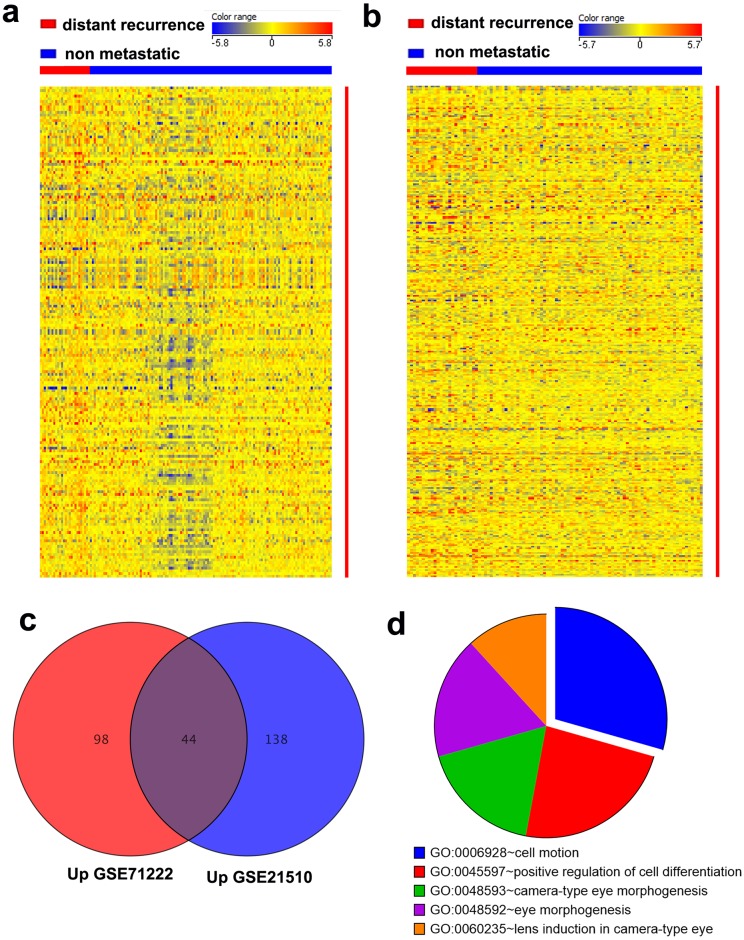
Genes associated with CRC distant recurrence (DR). Heatmap depicting the expression levels of differentially expressed genes (1.5 fold changes and *P* ≤ 0.05) between DR and non-recurrent (NR) CRC patients from the GSE71222 **(a)** and GSE21510 **(b)** datasets. Each column represents an individual sample and each row represents a single transcript. The expression level of each mRNA in a single sample is depicted according to the color scale. **(c)** Venn diagram depicting the common upregulated genes between DR and NR CRC samples from the GSE71222 and GSE21510 datasets. **(d)** Pie chart illustrating the distribution of the top 5 pathway designations for the 44 common upregulated transcripts from (c). The pie size corresponds to the number of matched entities.

To devise a gene expression panel associated with CRC DR with high confidence, we analyzed two independent CRC gene expression datasets (GSE71222 and GSE21510) and identified the genes associated with patient recurrence. Analysis of the GSE71222 and GSE21510 datasets revealed 180 (1.5 FC, *P* < 0.05) and 317 (1.5 FC, *P* < 0.05) differentially expressed transcripts between DR and non-metastatic tumors, respectively (Fig 1a and 1b). To identify DR-related genes with high confidence, we crossed the differentially expressed genes from the two datasets that revealed 44 common upregulated transcripts, comprising 37 genes (Fig 1c, Table 2), and three common downregulated genes (Table 2). Pathway analysis performed on the common upregulated genes revealed enrichment in several cellular pathways, including cell motion and regulation of cell differentiation (Fig 1d).

**Table 2:** Common recurrence-related genes in the GSE71222 and GSE21510 datasets.

Gene Symbol	FC (GSE71222)	FC GSE21510
**Upregulated genes**		
*LAMC2*	1.51	1.60
*SERPINA3*	1.58	2.33
*LPL*	1.74	2.42
*S100A2*	1.79	2.12
*PROM1*	1.99	2.33
*COL9A3*	1.77	2.13
*SERPINB5*	1.85	2.57
*TNFRSF11B*	2.08	2.28
*TCN1*	2.15	2.73
*C4BPA*	1.60	2.12
*SLC14A1*	1.50	1.80
*REG1B*	2.50	2.42
*VIP*	1.67	2.06
*HOXC6*	1.75	2.50
*MSX2*	1.56	1.63
*BMP4*	1.50	1.60
*TNIK*	1.62	1.56
*PRUNE2*	1.71	1.66
*KRT6B*	1.90	3.45
*NOV*	1.62	1.73
*TESC*	1.71	1.83
*DACT1*	1.52	1.72
*BHLHE41*	1.60	2.06
*ABHD2*	1.59	1.58
*AMIGO2*	1.90	1.87
*DCDC2*	1.82	2.18
*CD109*	1.67	1.86
*EPHA4*	1.80	2.32
*PPP2R2C*	1.71	1.85
*SOX2*	1.58	1.82
*EPHB1*	1.84	2.03
*GPR155*	1.72	1.72
*SBSPON*	1.86	1.93
*TMEM71*	2.16	2.91
*KIF26B*	1.97	1.52
*C3ORF70*	1.50	1.70
*CPA6*	1.56	1.76
**Downregulated genes**		
*PTPRD*	-2.09	-2.04
*PID1*	-1.52	-1.54
*ELF5*	-1.67	-1.62

Selected genes are based on a fold-change (FC) of 1.5 and *P* < 0.05 cut-off threshold.

### Validation of the DR-associated gene panel in the TCGA CRC dataset

**Fig 2:**
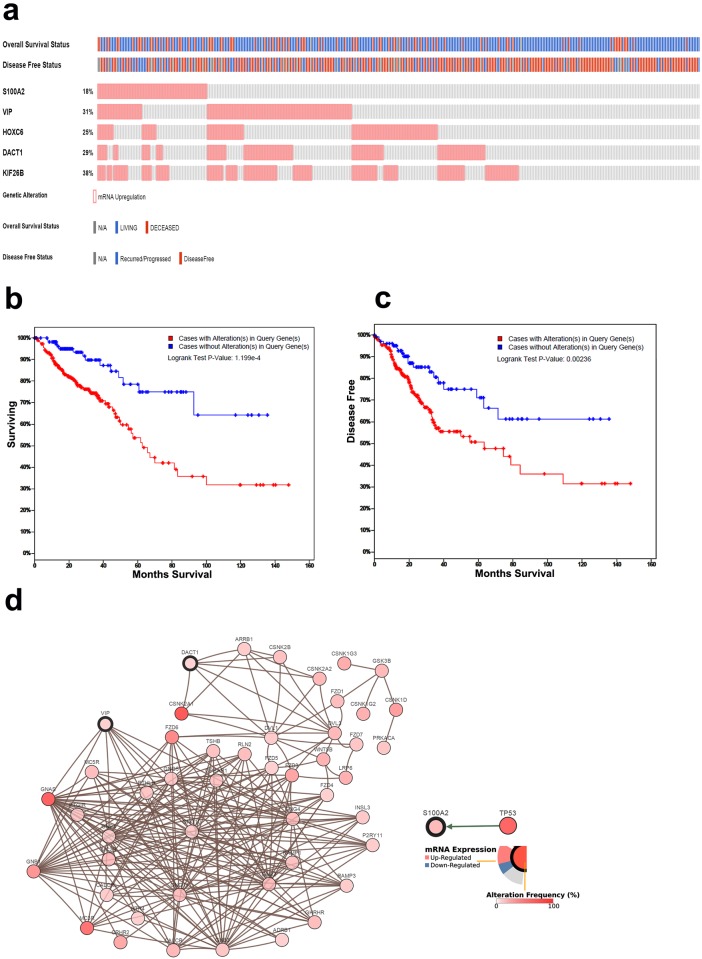
Validation of the distant recurrence (DR) gene panel in the TCGA dataset. **(a)** OncoPrint of the DR five-gene signature in the TCGA CRC dataset. Alteration in the expression of different members of the five-gene signature (rows) in relation to each sample (columns). Relationships to overall and disease-free survival are also shown. CRC cases with upregulated expression of the DR signature showed worse overall **(b)** and disease-free **(c)** survival than cases with lower expression. **(d)** Network view of the *VIP/DACT1/S100A2* neighborhood in CRC. *VIP*, *DACT1*, and *S100A2* are seed genes (indicated with thick borders), and all other genes were identified as altered in CRC.

We subsequently focused on the potential role of the upregulated genes in CRC recurrence. Therefore, each of the 37 upregulated genes was further validated using the TCGA CRC dataset to determine their relationship to overall survival (OS) and disease-free survival (DFS). *S100A2*, *VIP*, *HOXC6*, *DACT1*, and *KIF26B* were significantly associated with OS (*P*≤0.01) and DFS (*P*≤0.05), while *LAMC2*, *NOV*, and *AMIGO2* were only associated with DFS (*P*≤0.05). We subsequently focused on the five-gene panel that was associated with OS and DFS. The OncoPrint for this gene panel in the TCGA CRC dataset with the proportion of patients overexpressing each gene is presented in Fig 2a. Interestingly, the combination of this five-gene panel revealed a higher prognostic value, in which patients overexpressing at least one of the five genes showed a worse OS (log-rank test *P*-value: 1.19 × 10^−4^, Fig 2b) and worse DFS (log-rank test *P*-value: 0.002, Fig 2c) than those with lower expression of these genes. Data from the univariate analysis were subsequently put into the Cox proportional hazards multiple regression model to identify the independent factors for prognosis. The results showed that expression of the five-gene panel and tumor stage retained their significance as independent prognostic factors for CRC DFS and OS (p = 0.0023 and 0.0001 for DFS and p = 0.0086 and <0.0001 for OS, respectively), while age at diagnosis only correlated with OS, p = 0.0004 (Table 3). Network analysis of this five-gene signature revealed multiple network interactions in CRC, such as between *VIP* and *GNG11*, *GNB3*, *GNG12*, *GNB2*, *GNG5*, *GNAS*, *GNG2*, *GNB4*, *GNG4*, *GNG10*, and *GNB1*; between *DACT1* and *ARRB1*, *DVL1*, *CSNK2B*, *CSNK2A1*, and *CSNK2A2*; and between *S100A2* and *TP53* (Fig 2d).

**Table 3:** Multivariate analyses for the prognostic value of the 5-gene signature in TCGA CRC dataset.

Parameters	Categories	DFS hazard ratio (95% CI)	P value	OS hazard ratio (95% CI)	P value
Five-gene expression	High vs Low	1.95 (1.27 to 3.01)	**0.0023**	1.84 (1.16 to 2.90)	**0.0086**
Age at diagnosis	<65 vs. >65	0.86 (0.56 to 1.33)	0.5103	2.47 (1.50 to 4.09)	**0.0004**
Type	CA vs RA vs MA	0.87 (0.67 to 1.12)	0.2925	1.14 (0.86 to 1.51)	0.3560
Tumor stage	(3/4 vs 1/2)	1.92 (1.37 to 2.69)	**0.0001**	3.18 (1.96 to 5.16)	**<0.0001**
Gender	M vs F	1.35 (0.86 to 2.10)	0.1857	1.04 (0.65 to 1.65)	0.8614

CA: Colon Adenocarcinoma; RA: Rectal Adenocarcinoma; MA: Mucinous Adenocarcinoma of the Colon and Rectum

## Discussion

In the current study, we retrospectively derived and validated a gene expression signature associated with the risk of systemic relapse in patients with CRC. Analysis of the GSE71222 and GSE21510 datasets identified 37 upregulated and three downregulated genes associated with DR in CRC. Interestingly, several of the identified genes (*LAMC2*, *LPL*, *SERPINB5*, *TCN1*, *VIP*, *MSX2*, *PRUNE2*, *KRT6B*, *TESC*, *EPHA4*, *GPR155*, *KIF26B*, *C3ORF70*, and *PID1*) were also found to be differentially expressed in our previous global mRNA expression profiling of CRC compared to adjacent normal mucosa, suggesting a plausible role of these genes in driving CRC in addition to DR [16]. Concordant with our data, Takahashi and colleagues [11] reported a worse prognosis in CRC patients overexpressing Traf2- and Nck-interacting kinase (*TNIK*). Higher expression of *MSX2* was found to be associated with metastasis in different types of human cancers [17]. *PROM1*, also known as *CD133*, was among the 37 upregulated genes in both datasets. Interestingly, *PROM1* has previously been reported as a cancer stem cell marker in CRC [18, 19]. Similarly, two of the identified genes in the current study (*SLC14A1* and *KIF26B*) were identified in an intestinal stem cell signature previously reported to be associated with poor clinical outcome in CRC [20]. Therefore, it is possible that patients with an enriched CSC phenotype are more likely to develop DR. We subsequently validated this gene signature in the TCGA CRC dataset, which includes 379 patients. Our analysis narrowed down the CRC recurrence signature to five genes (*S100A2*, *VIP*, *HOXC6*, *DACT1*, and *KIF26B*) whose expression was associated with poor OS (log-rank test *P*-value: 1.19 × 10^−4^) and DFS (log-rank test *P*-value: 0.002), which was further confirmed in a multivariate analysis. Therefore, we here present a novel gene expression signature for predicting the risk of systemic relapse in CRC. Concordant with our data, overexpression of *S100A2* has been associated with poor clinical outcome in colorectal [21] and oral [22] cancers. The *HOXC6* gene is frequently upregulated in prostate cancer, although no association with patient relapse was observed [23]. *DACT1* was recently shown to promote CRC tumorigenicity and invasion via stabilization of β-catenin [24]. Concordantly, overexpression of *DACT1* was observed during the transition of ductal carcinoma *in situ* to invasive ductal carcinoma in breast cancer [25].

## Conclusion

Herein, we integrated multiple gene expression datasets and devised a novel five-gene signature as an independent predictor of CRC DR. This signature adds to the current prognostic value of tumor staging. Before this five-gene-signature can be utilized in the clinic; however, additional validations are required
